# Spectrum of Imaging Findings in Paget’s Disease of the Breast—A Pictorial Review

**DOI:** 10.1007/s13244-015-0415-z

**Published:** 2015-07-05

**Authors:** Smiti Sripathi, Anurag Ayachit, Rajagopal Kadavigere, Sandeep Kumar, Asha Eleti, Aron Sraj

**Affiliations:** Department of Radiodiagnosis and Imaging, Kasturba Medical College, Manipal, Karnataka India; Breast Unit, Department of Radiodiagnosis, Southend University Hospital, Essex, UK

**Keywords:** Paget’s, Nipple, DCIS, Invasive, Malignancy

## Abstract

**Purpose:**

We aimed to demonstrate imaging features of Paget’s disease of breast, which is an extremely uncommon malignancy that presents with changes in the nipple-areolar region that may or may not be associated with an underlying in situ component or invasive cancer.

**Methods and Results:**

Mammography is the initial investigation of choice, having a high sensitivity especially in cases where a palpable mass is present. The addition of ultrasound improves the accuracy of mammography. When both mammography and ultrasound are negative, MRI may detect an underlying mass or ductal carcinoma in situ (DCIS).

**Conclusion:**

The surgical management of Paget’s disease includes mastectomy with or without axillary dissection, though breast conservation surgery in the form of wide local excision can also be done in a selected group of patients. Management should be based on both clinical and imaging findings, including mammography and ultrasound, with MRI playing a crucial role in defining the extent of involvement.

*Teaching Points*

• *To differentiate Paget’s disease from other chronic skin conditions.*

• *Mammographic and ultrasound findings of histopathologically established Paget’s disease.*

• *When ultrasound and mammogram are negative, MRI may detect underlying malignancy.*

## Introduction

Paget’s disease of breast is a rare malignancy accounting for 1–3 % cases of breast cancer [1] and was first described by Sir James Paget in 1874 [2]. Almost 80–90 % of cases are associated with underlying malignancy in the form of ductal carcinoma in situ (DCIS) or invasive breast cancer. The surgical management is individualized and is based on imaging and histopathological findings. In this pictorial essay, we describe the spectrum of imaging findings in biopsy proven cases of Paget’s disease of breast.

### Discussion

Paget’s disease is an unusual breast malignancy where clinical features may be the only sign of cancer during the initial presentation. Imaging plays an important role in evaluating the extent of involvement and in deciding upon patient management.

### Clinical Findings

Paget’s disease of breast is commonly seen in the fifth or sixth decade of life, although it can also be seen in adolescents and the elderly [3]. It is more common in males compared to females [4] and is usually unilateral [5]. The patients present clinically with changes in the nipple and areolar region in the form of erythema, ulceration, eczematous changes and induration of skin that may be associated with nipple retraction and blood stained nipple discharge [1]. Some of these patients may first approach a dermatologist for treatment of eczema. In cases presenting only with eczema, there is an initial phase of clinical improvement with symptomatic treatment, which causes a delay in diagnosis [6]. A biopsy from the nipple areolar region is necessary to confirm the diagnosis. The differential diagnosis includes allergic contact dermatitis, irritant dermatitis, lichen simplex chronicus and psoriasis [7]. Advanced cases of Paget’s disease may present with a well-demarcated round or ovoid eczema-like plaque that is easily distinguished from the surrounding normal skin. In some cases, a palpable mass may be felt clinically, which is important since approximately 90–95 % of these patients have an underlying invasive cancer [8]. One must not confuse cases of Paget’s disease with inflammatory carcinoma in which there is diffuse erythema involving the entire breast with secondary involvement of nipple and areola.

### Etiopathogenesis

There are two important theories that explain the possible pathogenesis of Paget’s disease.

#### Epidermotropic Theory

**Fig. 1:**
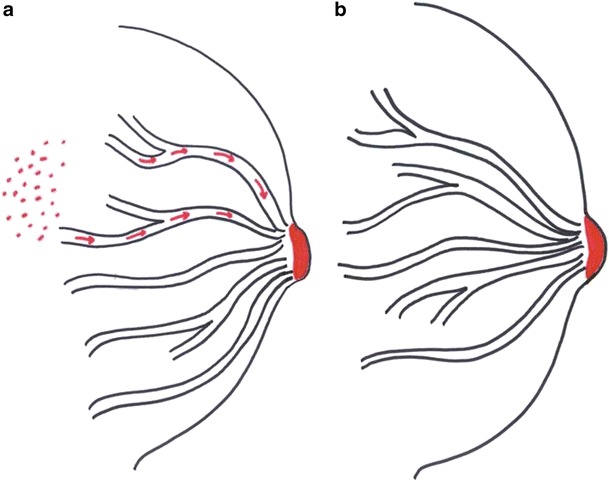
Line diagram showing Etiopathogenesis theories. **a** Epidermotropic theory: Malignant cells from DCIS migrate through the lactiferous ducts to reach the nipple and areola. **b** Intraepidermal transformation theory: Paget cells arise from the epidermis of nipple by degeneration of existing cells or by in-situ transformation

According to this theory, malignant cells from DCIS migrate through the lactiferous ducts and reach the nipple and areola (Fig. 1a) [9]. This is one of the widely accepted theories as the Paget cells and the underlying carcinoma share the same immunohistochemical profile.

#### Intraepidermal Transformation Theory

According to this theory, Paget cells arise from the epidermis of nipple by degeneration of existing cells or by in-situ transformation (Fig. 1b). This is supported by the fact that in some cases of Paget’s disease, findings are confined to the nipple-areolar region with no underlying DCIS or invasive cancer.

## Imaging Features

What role does imaging have in the diagnosis of Paget’s disease? Usually, the patient is referred for imaging after a biopsy from the lesion, and this confirms the diagnosis of Paget’s disease. Mammography is the initial radiological investigation for detecting underlying invasive carcinoma or DCIS; however, it may be normal, and ultrasound is performed when mammogram is negative. MRI of breast is performed in patients with negative mammogram and ultrasound findings with no clinically palpable mass.

### Mammography

**Fig. 2:**
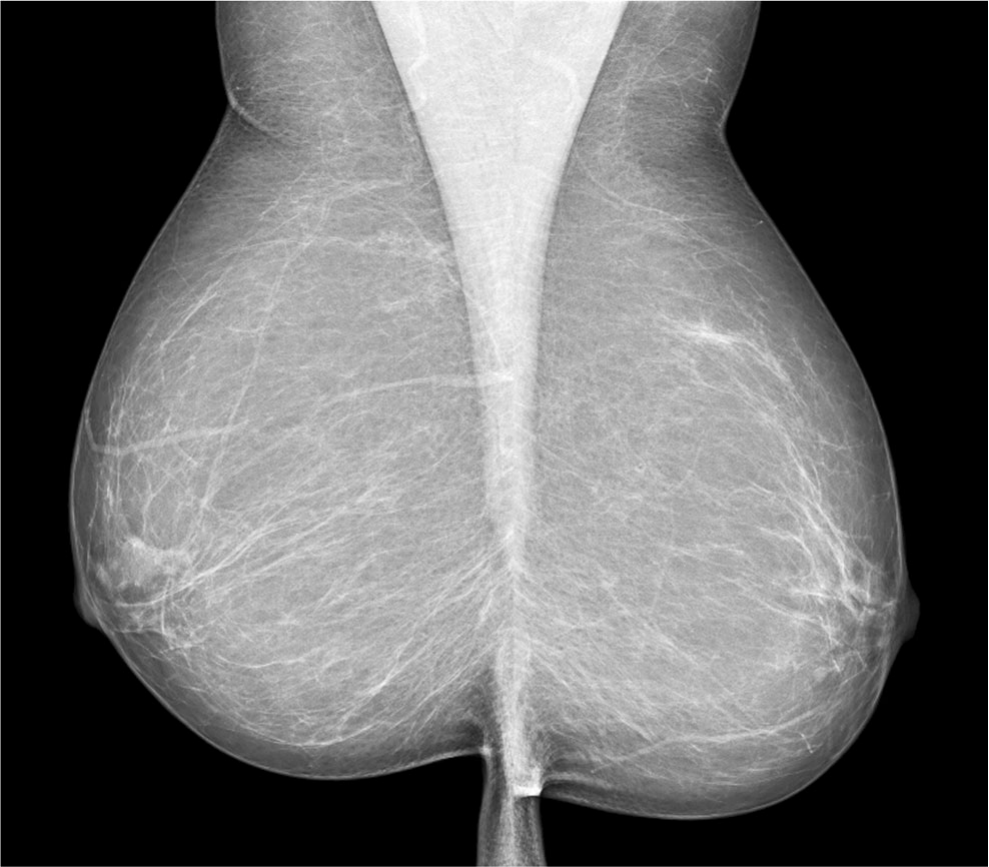
Normal mammogram (MLO view) in a 66-year-old female with eczema of right nipple which proved to be Paget’s disease of the nipple on histopathology. Patient underwent wide local excision

**Fig. 3:**
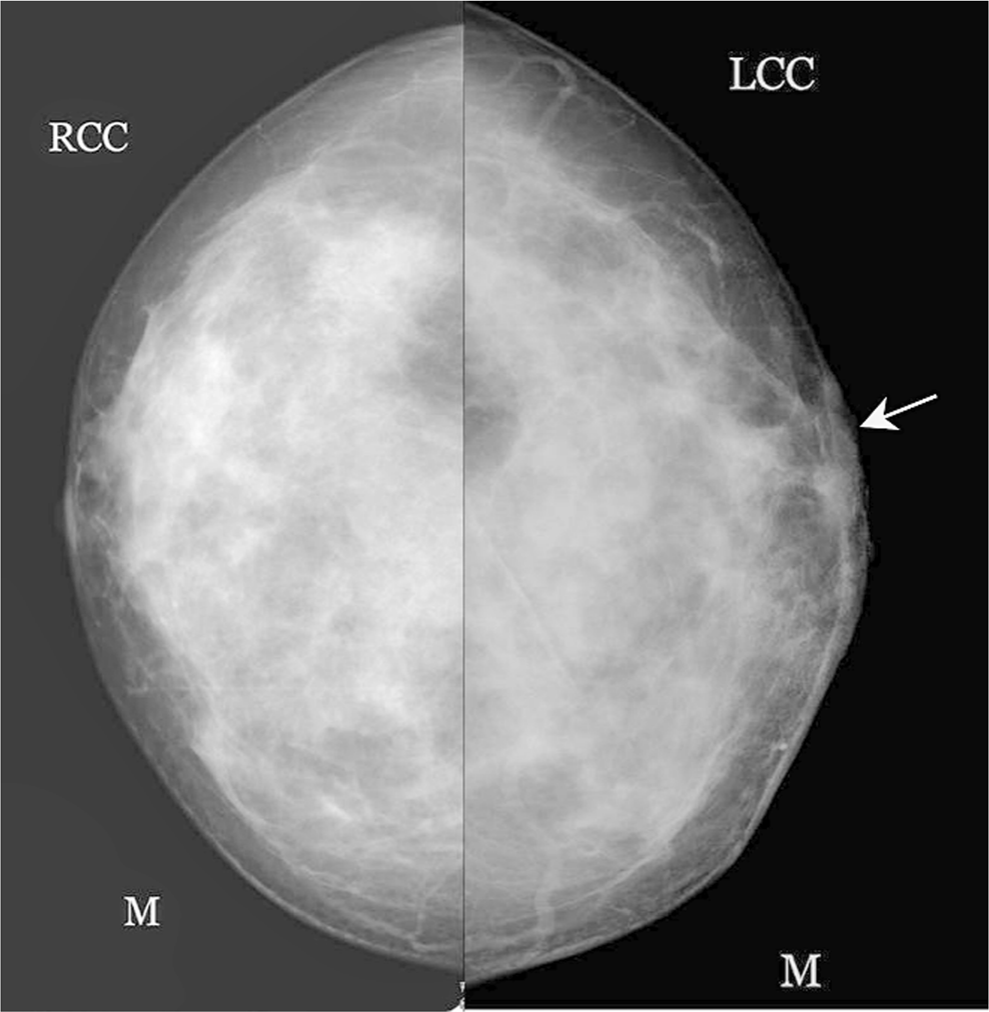
A 37-year-old female presented with ulcer over the left nipple and areolar region for 1 year with foul smelling nipple discharge, pain and pruritis. Mammogram of left breast (Fig. 3) showed skin thickening (*arrow*) in nipple and areolar region. The patient underwent central quadrentectomy and no mass was found in the underlying breast. Follow-up mammogram after 1 year was normal

**Fig. 4:**
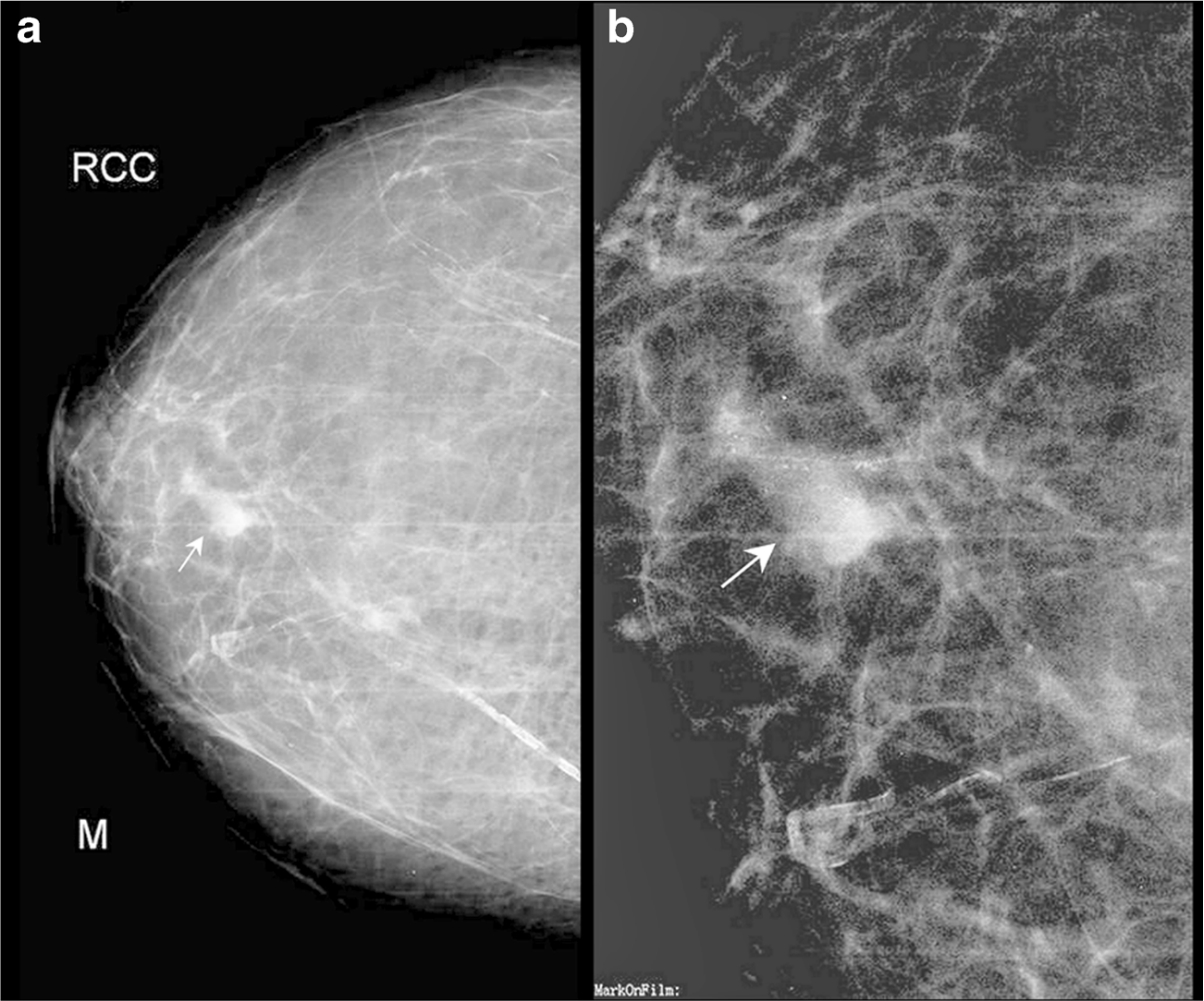
A 71-year-old female came with complaint of itching and eczema around the right nipple with a history of nipple discharge. There was no mass palpable on clinical examination. Mammogram of right breast showed an asymmetric density (*arrow*) in cranio-caudal (CC) view (**a and b**) that was not seen on ultrasound. The patient underwent simple mastectomy. Histopathology showed features of Paget’s disease of the nipple with invasive ductal carcinoma grade 2

**Fig. 5:**
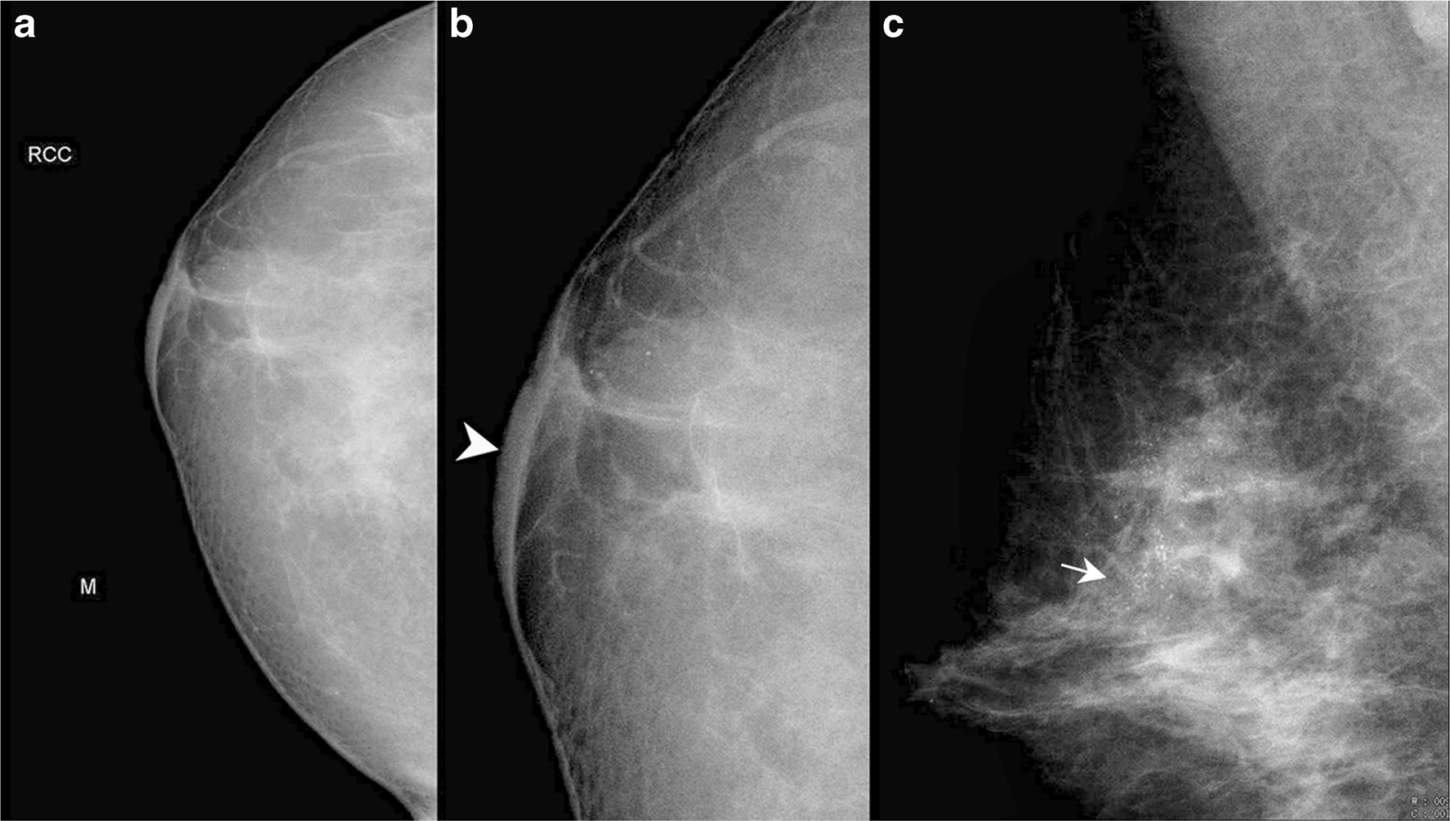
A 50-year-old female presented with history of nipple retraction and erythematous scaly plaque over the right nipple areolar region, along with blood stained nipple discharge for 6 months. Mammogram of the right breast showed skin thickening (*arrowhead*) – CC view (**a**), with an ill-defined density in upper quadrant with clustered and pleomorphic calcifications (*arrow*) – MLO view (**b**). The patient underwent simple mastectotomy. Histopathology showed features of Paget’s disease of the nipple with clinging DCIS

**Fig. 6:**
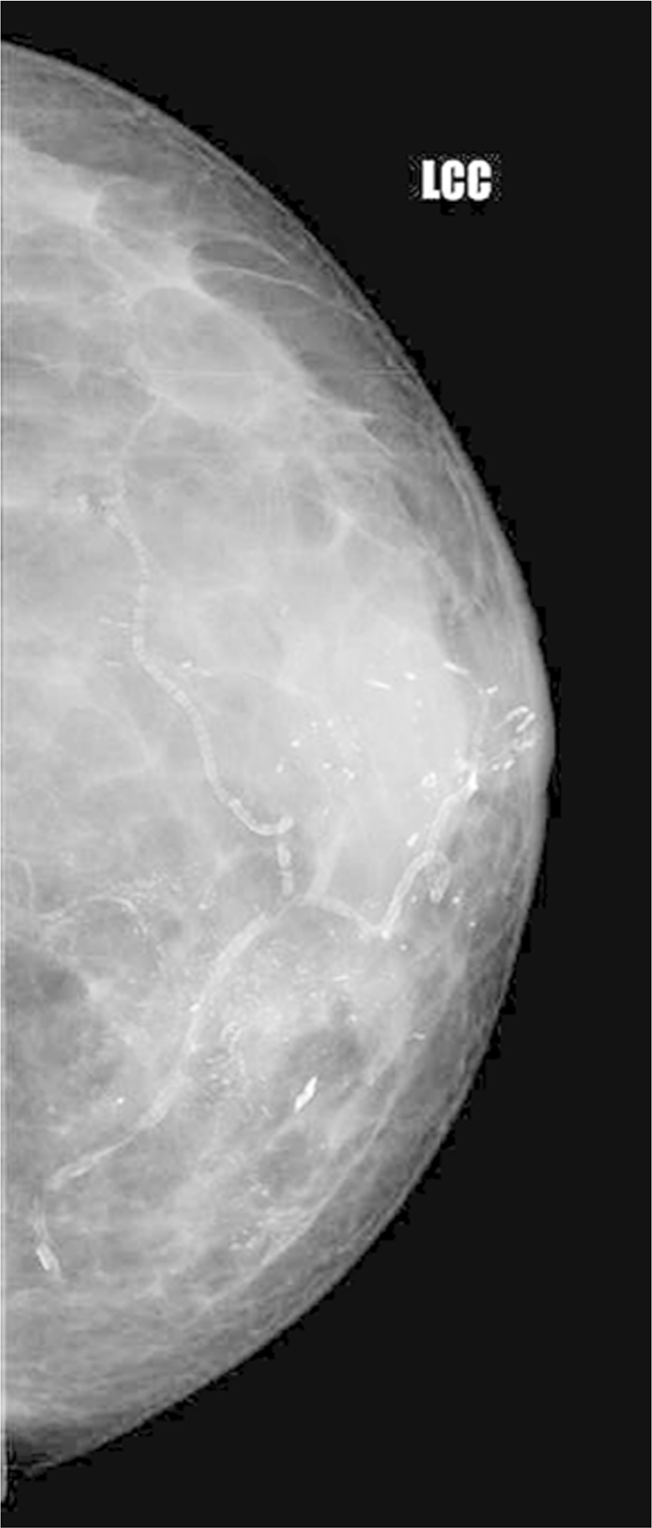
A 43-year-old female with history of ulceration of the left nipple and watery discharge for 2 years underwent biopsy of the nipple, which showed features of Paget’s disease. Mammogram of the left breast showed multiple pleomorphic calcifications in a segmental distribution, suggestive of DCIS. The patient underwent modified radical mastectomy

Mammography plays an important role in the diagnosis and management of Paget’s disease; however, it has its own limitations and may be normal in some cases (Fig. 2). The common mammographic findings are skin thickening in the nipple areolar region (Fig. 3), asymmetric density (Fig. 4), nipple retraction or a discrete mass. The mass may be sub-areolar or distant from the nipple-areolar region. Other findings include malignant pleomorphic calcifications (Figs. 5 and 6) that may or may not be associated with skin thickening or architectural distortion. The role of mammography in Paget’s disease is not only in detection of an underlying mass or DCIS, but also in follow-up of patients undergoing conservative breast surgery so that recurrence can be ruled out. Various studies have evaluated the role of mammography in Paget’s disease. In a study by Gunhan-Bilgen et al. [10], 15 % of cases had a negative mammogram while Ikeda et al. concluded that 50 % cases with typical clinical features of Paget’s disease had negative mammography findings [11]. Morrogh et al. found that 65 % patients with a negative mammogram had an underlying unifocal cancer [12]. Muttarak et al. [13] studied the clinical, imaging and pathological findings in 16 cases of Paget’s disease and found a high degree of invasive carcinoma as most of their patients presented late and had positive mammographic findings.

### Ultrasound

**Fig. 7:**
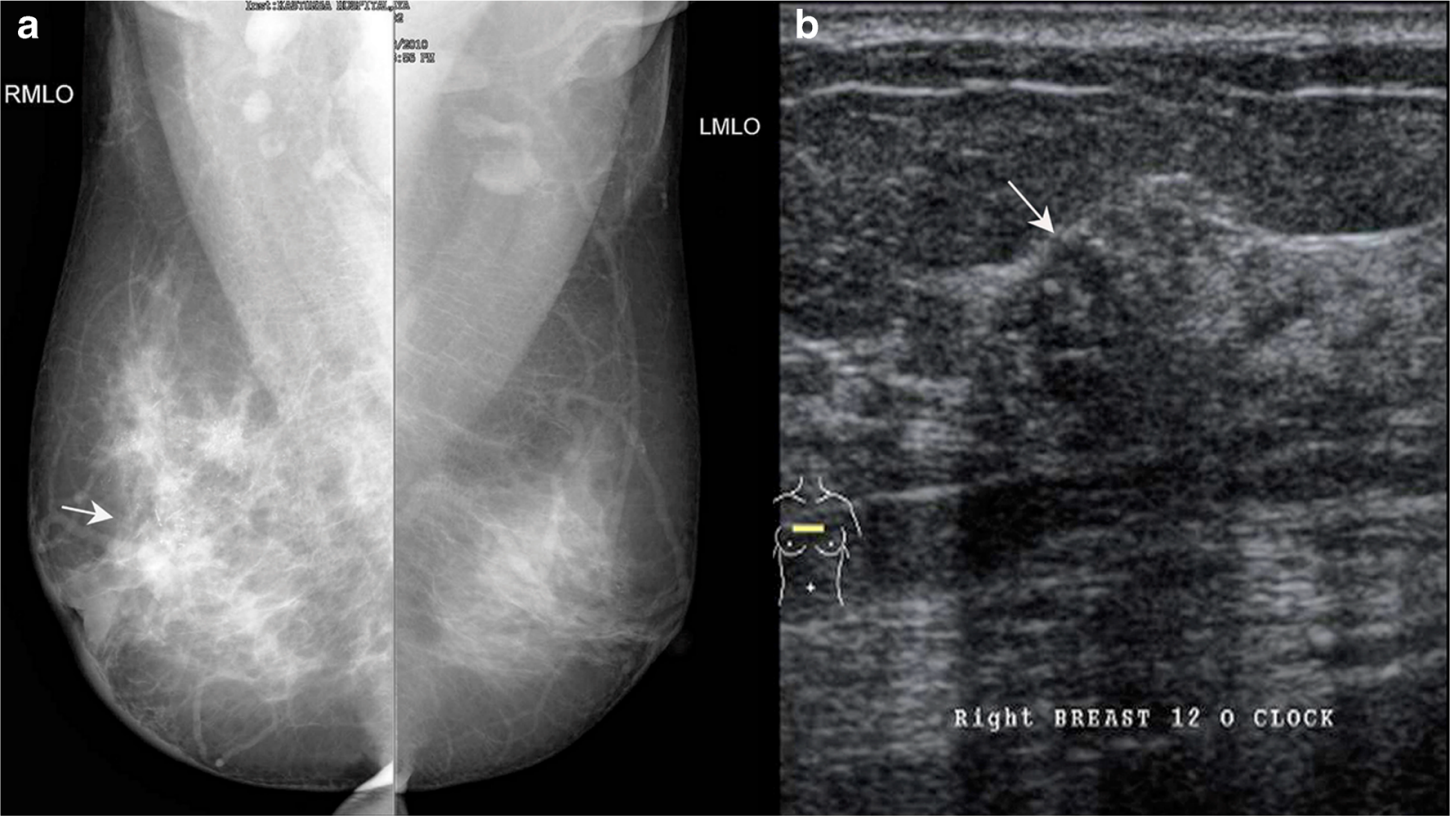
A 37-year-old female presented with right nipple retraction and discharge for 8 months with pain, swelling and crusty skin along the nipple and areolar region. Biopsy from the nipple areolar region was suggestive of Paget’s disease. Mammogram of the right breast in MLO view (**a**) showed multiple clustered foci of microcalcification (*arrow*). Ultrasound showed a small ill-defined hypoechoic lesion (**b**) measuring 1.2 × 1 cm at the 12 o’ clock position of the right breast with posterior acoustic shadowing. The patient underwent a simple mastectomy of the right breast and histopathology revealed Invasive carcinoma

**Fig. 8:**
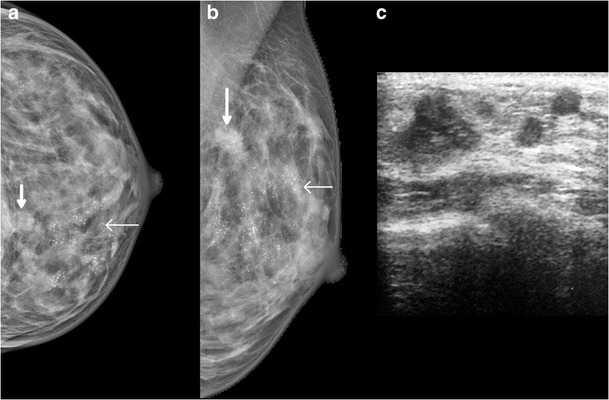
A 49-year-old female came with the complaint of discharge from the left nipple. Mammogram of left breast, MLO and CC view (**a** and **b**), shows extensive pleomorphic calcifications (*arrow*) with mass (*arrowheads*) in the upper and inner quadrant. Ultrasound of the left breast (**c**) shows an irregular hypoechoic lesion (*arrow*) in the inner and upper quadrant of the left breast, with two similar smaller lesions adjacent to it. The patient underwent left mastectomy and histopathology showed features of Paget’s disease of the nipple with infiltrating ductal carcinoma and high grade DCIS

**Fig. 9:**
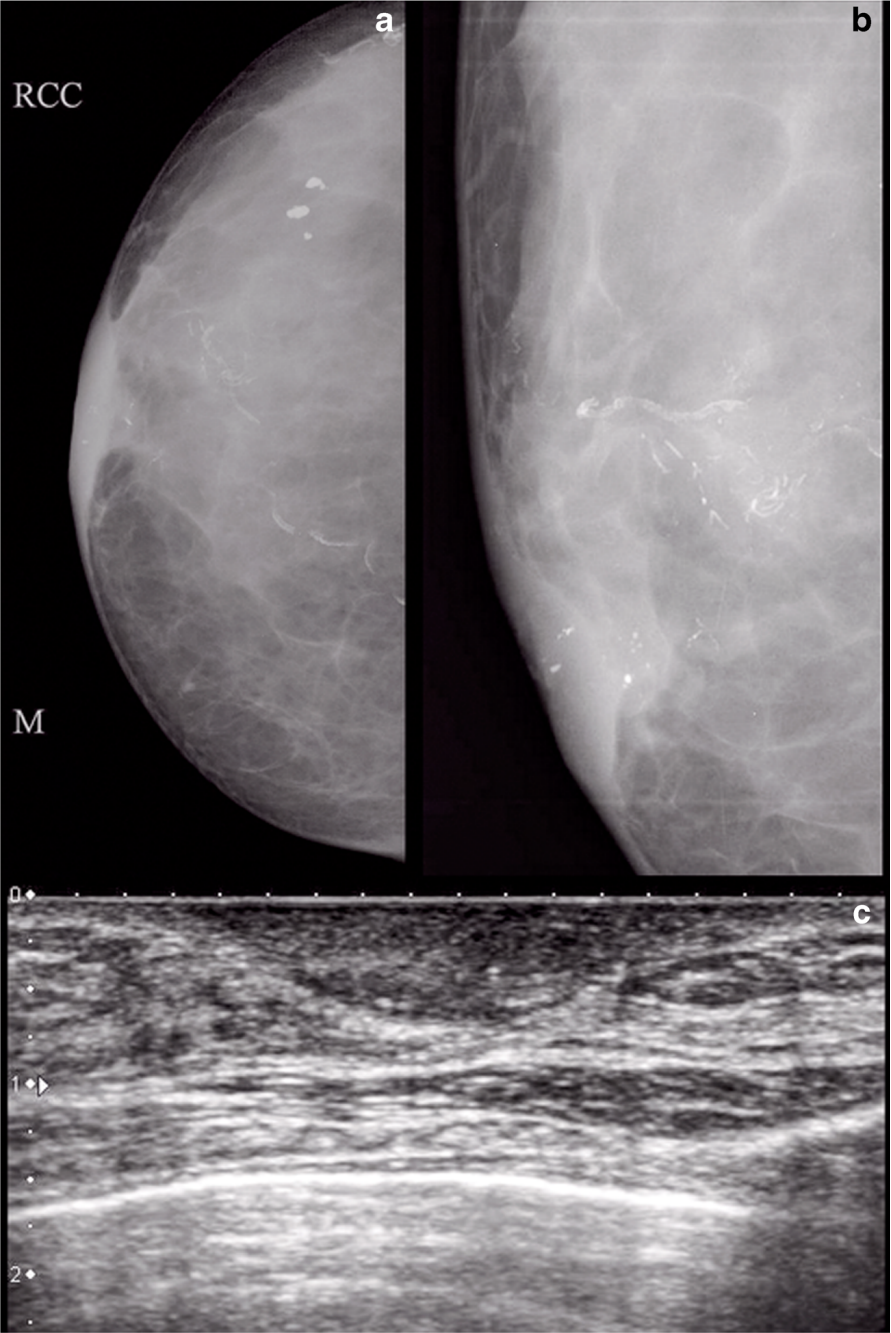
A 62-year-old female presented with history of nipple discharge from the right breast for 2 months, along with a palpable lump in near the nipple region. Mammogram of right breast, CC view (**a**) and magnified view (**b**), shows skin thickening in the nipple-areolar region with pleomorphic calcification. Ultrasound (**c**) of the right breast showed a well-defined heterogenous thickening below the nipple–areolar region. The histopathology was suggestive of Paget’s disease of breast with underlying DCIS and foci of microinvasion. The patient underwent wide local excision and radiotherapy

Ultrasound is used not only to confirm the mammography findings in a case of Paget’s disease, but also when the mammogram is negative. The findings on ultrasound include heterogeneous hypoechoic areas in breast parenchyma, presence of a discrete mass or dilated ducts. Microcalcifications are not appreciated on ultrasound. Sometimes, there may be an area of DCIS with pleomorphic calcification on mammogram with an underlying mass, which is better appreciated on ultrasound (Figs. 7a and b and 8a and b). In some cases, however, even when there is pleomorphic calcification with nipple areolar thickening on mammogram, ultrasound findings can be non-specific and may show only skin thickening in the nipple areolar region (Fig. 9a, b and c).

### Magnetic Resonance Imaging

**Fig. 10:**
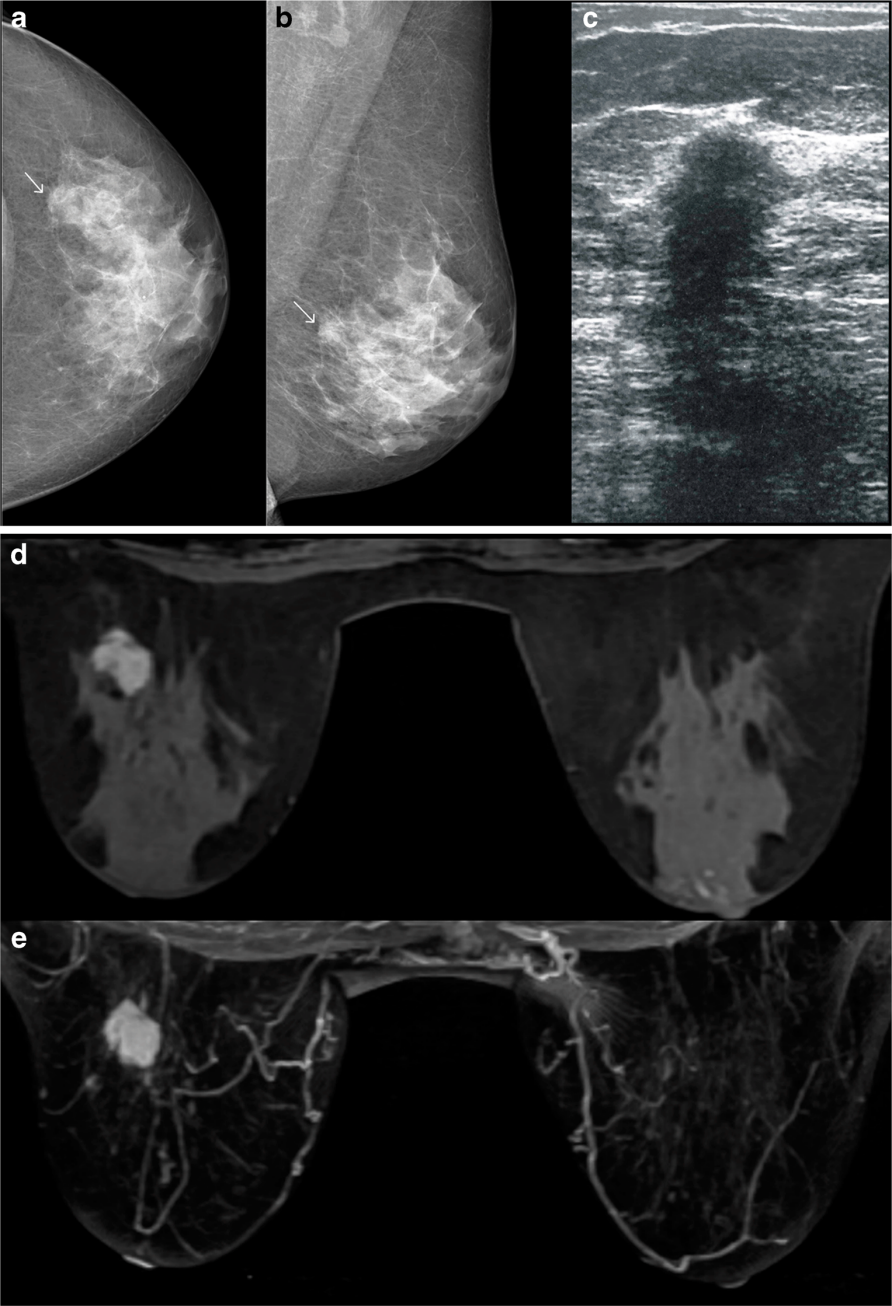
**a**, **b**, **c** A 68-year-old female presented with mild thickening along the nipple areolar region on the left. She underwent mammogram, which revealed an irregular mass (*arrows*) in outer and central region of left breast, CC and MLO view. **a** and **b**. Ultrasound of the left breast (**c**) shows an irregular hypoechoic lesion at the 2 o’clock position measuring 1.7cm in the long axis. **d** and **e** MRI of the breast of the same patient in the Fig. above. STIR (**d**) and T1W contrast sequences (**e**) show an enhancing irregular mass in the outer half of the left breast with focal enhancement along the nipple areolar region. The patient underwent left mastectomy with axillary clearance. The histopathology was suggestive of Paget’s disease of the nipple with underlying DCIS of solid and micropapillary growth pattern having a high nuclear grade

**Fig. 11:**
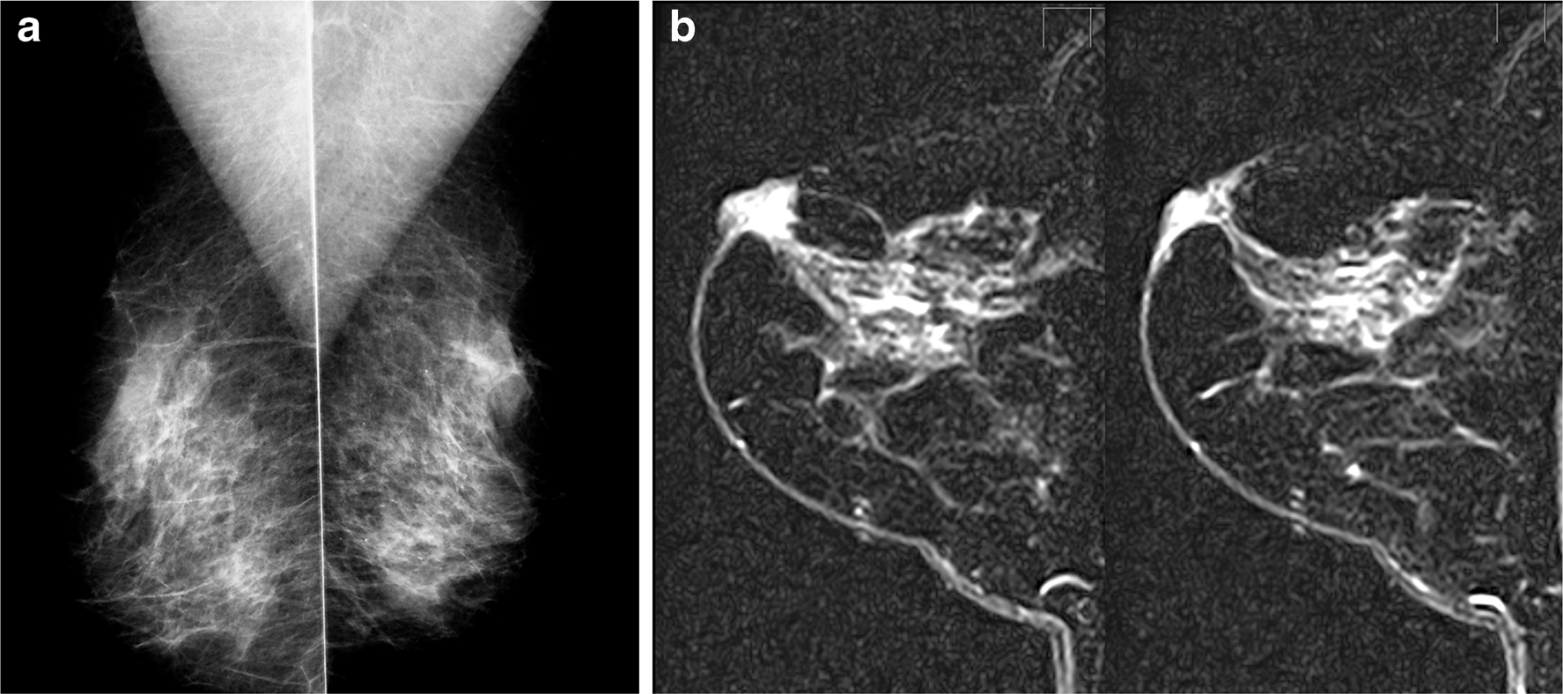
A 47-year-old patient presented clinically with minimal thickening in thenipple areolar region in the left breast. Mammogram (MLO view) of both breasts (**a**) was normal, while MRI of the left breast (**b**) showed enhancing thickening in nipple areolar region with no obvious underlying mass

With advances in technology and use of dedicated breast coils, magnetic resonance imaging (MRI) is increasingly being used for evaluation of clinically suspected cases of Paget’s disease (Figs. 10a–e and 11a and b). It is a highly sensitive modality and can be used in cases where both mammography and ultrasound findings are negative.

**Fig. 12:**
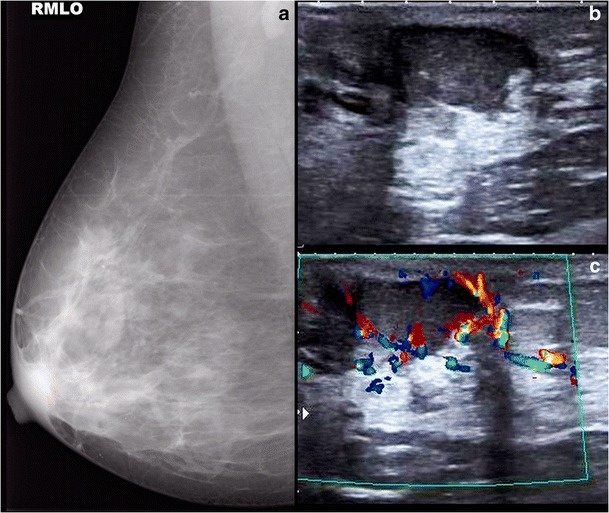
A 51-year-old female presented with a lump in the right nipple–areolar region of 3 months duration with an off-and-on history of nipple discharge. No history of fever was present. Mammogram of the right breast, MLO view (**a**), shows diffuse thickening in the nipple areolar region; however, no microcalcifications are seen. Ultrasound of the right breast (**b**) shows a well-defined hypoechoic lesion in the nipple areolar region with peripheral vascularity (**c**) on colour doppler

**Fig. 13:**
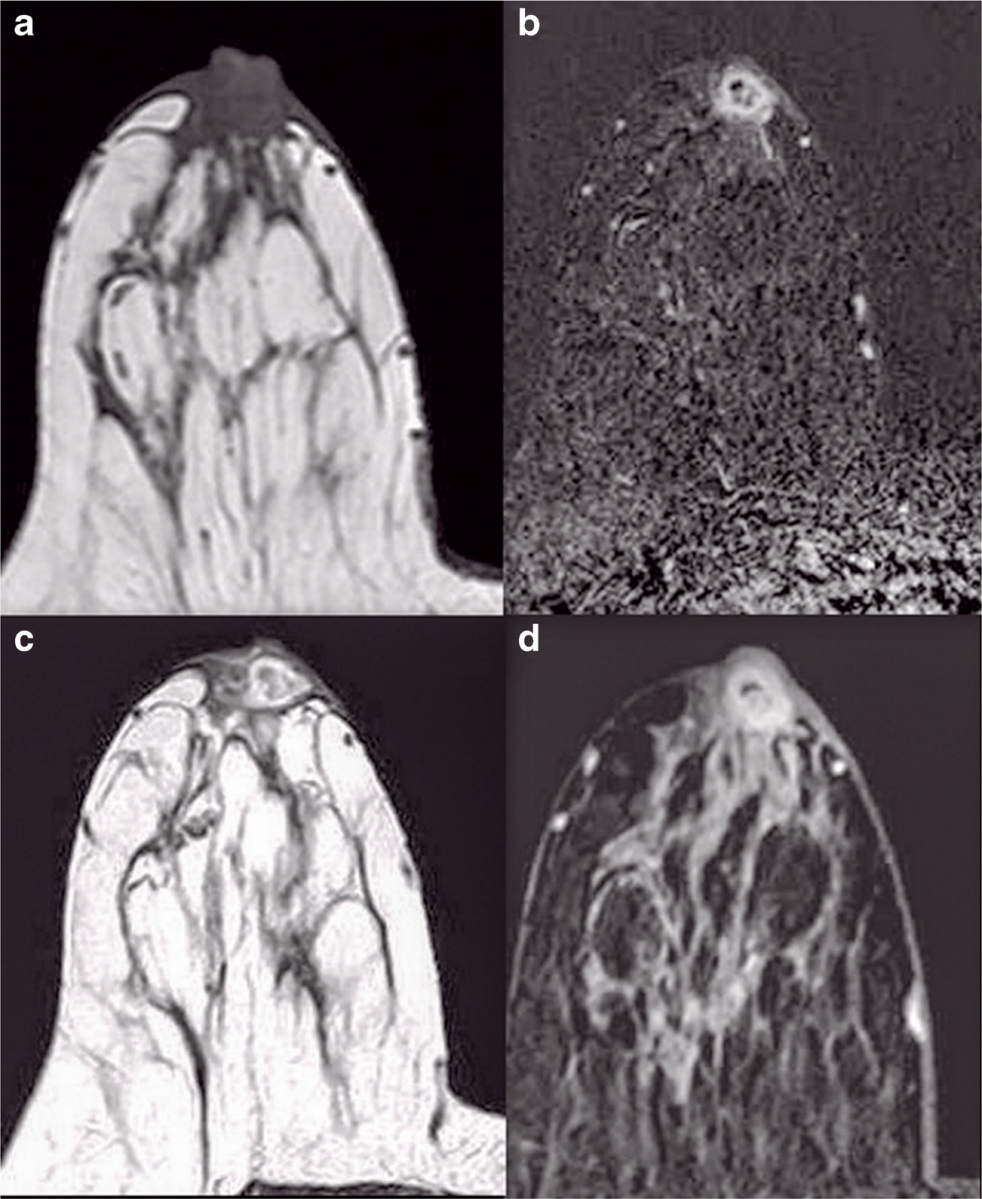
MRI of the right breast of the same patient as in Fig. 12 shows an irregular T1W isointense (**a**) lesion that is hyperintense on STIR (**b**) and T2W (**c**) with central hypointense area and peripheral enhancement on post-contrast T1W sequence. Biopsy from the nipple areolar region was suggestive of chronic sub-areolar abscess. The patient improved on oral antibiotics

At times there is a diagnostic dilemma in a case of chronic mastitis presenting with a thickening and palpable mass in the nipple areolar region, where imaging helps in making a diagnosis (Figs. 12 and 13) and helps in deciding management.

In a case report by Amano et al. [14], MRI accurately depicted underlying DCIS in a patient without a palpable mass or any positive mammography findings.

The findings on MRI include thickening and enhancement of nipple-areolar complex, an underlying mass, enhancing DCIS or a combination of these findings. Enhancement patterns of the normal nipple-areolar complex are variable, including absent, mild or intense enhancement [7]. However, on comparing the two sides, asymmetric enhancement is noted in the nipple-areolar region with the involved region showing irregular, discoid or irregular enhancement in the case of Paget’s disease [15]. Nipple involvement may also be seen even when there is no clinical suspicion of Paget’s disease [16]. If there is no mass in the sub-areolar region, the entire breast has to be examined to detect any mass that is away from the nipple-areolar region, as the management in such cases is based on imaging findings. Echevarria et al. [17] discussed the effectiveness of MRI in detecting occult breast cancer associated with Paget’s disease of the nipple-areolar complex, while Peters et al. [18] discussed the role of MRI as a problem-solving tool in patients with a normal mammogram and no clinically palpable mass. Frei et al. [19] evaluated pre-operative mammography and MRI findings in nine biopsy proven cases of Paget’s disease and concluded that Paget’s disease of breast with underlying DCIS can be effectively diagnosed by MRI. Though Paget’s disease is predominantly a clinical diagnosis, negative mammography cannot reliably exclude underlying malignancy. Both clinical and imaging findings are complementary to each in order to make a diagnosis [20].

### Management

The treatment of Paget’s disease has been controversial and depends upon the origin of Paget cells. If the epidermotropic theory is believed, then there is an invasive component in the breast and the treatment of choice is mastectomy. The intraepidermal transformation theory, however, says that Paget’s cells form de novo in nipple epithelium, and hence, the treatment of choice in cases confined to the nipple areolar region is resection of nipple-areolar complex followed by local irradiation, making the role of mastectomy controversial.

**Fig. 14:**
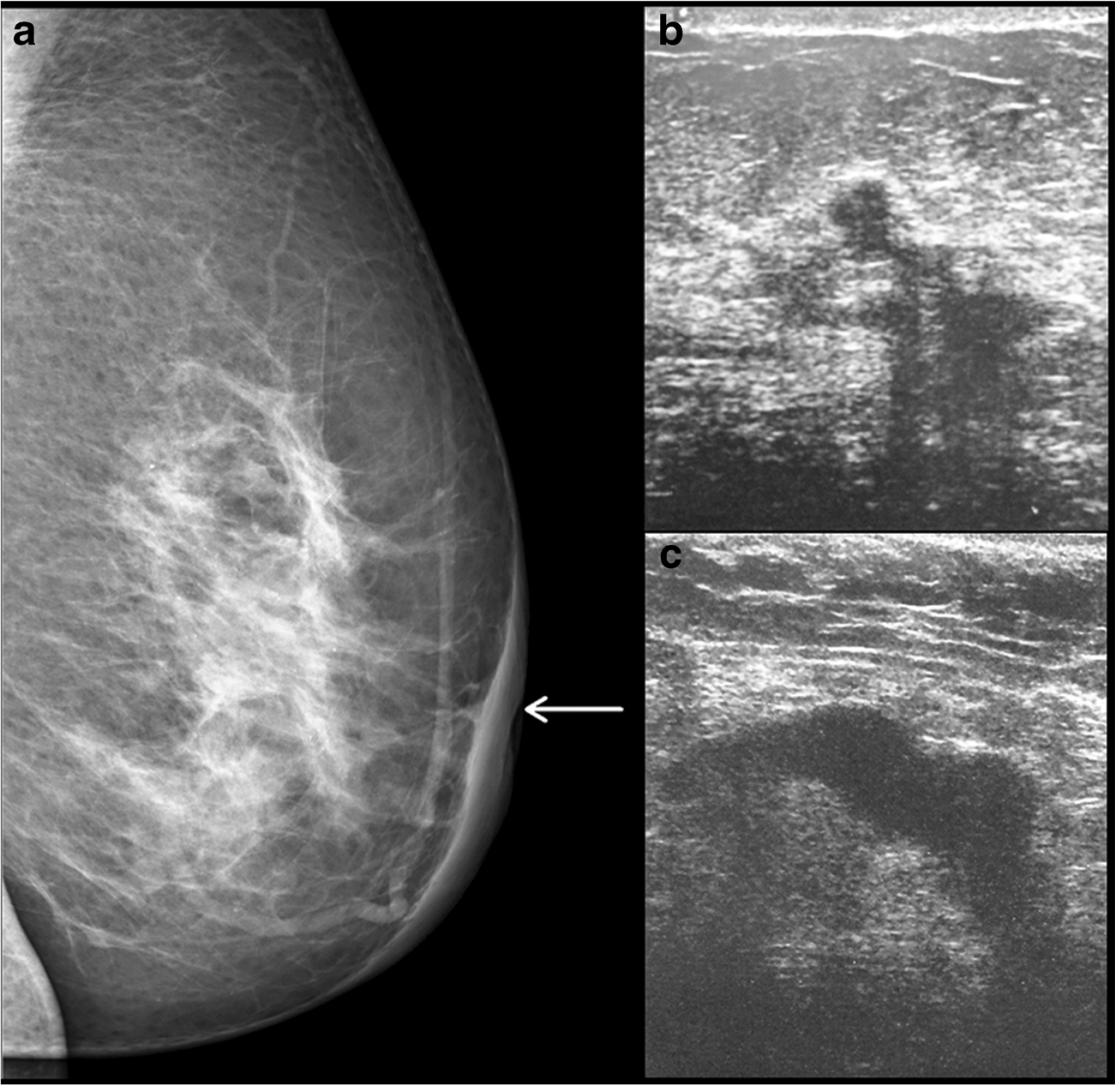
A 60-year-old female presented with complaint of ulceration and inversion of the left nipple, which showed features of Paget’s disease on histopathology. Mammogram of left breast, CC view (**a**), shows thickening of skin along the nipple areolar region, and ultrasound (**b**) revealed an ill-defined hypoechoic lesion in the left breast with enlarged lymph nodes in left axilla (**c**). The patient underwent left mastectomy with axillary clearance and histopathology showed features of Paget’s disease of the nipple with DCIS and axillary lymph node involvement

Earlier, the treatment was mastectomy with or without axillary dissection. In one of the earliest studies by Joseph and Robinson in 1981, involvement of axillary lymph nodes was described as an important prognostic factor, and modified radical mastectomy was recommended in this group of patients [21]. There may be recurrence or distant metastasis in patients with axillary lymph node involvement (Fig. 14). Kothari et al. studied 70 patients of Paget’s disease and found that since it is often associated with underlying malignancy, cone excision of nipple would result in incomplete excision in 75 % cases [22]. Dixon et al. reported high recurrence rates (40 %) in cases treated with local excision where there was an in-situ component along the nipple region [23]. In cases of Paget’s disease of the nipple with DCIS or invasive cancer away from the nipple areolar region, complete resection of disease and the nipple areolar complex is needed, followed by radiotherapy [24]. Dalburg et al. did not find any difference in survival rates in patients of Paget’s disease who underwent breast conservative surgery or mastectomy [25]. Therefore, in a histopathology proven case of Paget’s disease of the nipple, a pre-operative MRI of the breast should be performed to know the exact nature and extent of disease, especially when breast conservative surgery is contemplated as the treatment.

## Conclusion

Paget’s disease of the breast is an uncommon malignancy that is associated with an underlying in situ component or invasive cancer in a large percentage of cases. Surgical management, i.e., breast conservative surgery versus mastectomy, should be decided based on both clinical and imaging findings, including mammography and ultrasound, with MRI playing a crucial role in defining the extent of involvement.
